# Lymphome malin non hodgkinien du cavum: protocoles thérapeutiques et facteurs pronostiques

**DOI:** 10.11604/pamj.2015.22.153.7916

**Published:** 2015-10-16

**Authors:** Saloua Ouraini, Ismail Nakkabi, Fouad Benariba

**Affiliations:** 1 Service d'ORL et CCF, Hôpital Militaire d'Instruction Mohammed V, CHU Ibn Sina, Rabat, Maroc

**Keywords:** Cavum, obstruction nasale, lymphome, radio-chimiotherapie, Cavum, nasal obstruction, lymphoma, radio-chemotherapy

## Abstract

Le lymphome malin non hodgkinien est une entité histologique rare parmi les cancers du cavum, la plupart des tumeurs du nasopharynx étant des carcinomes indifférenciés ou Undifferencied Carcinoma of Nasopharyngeal Type (UCNT); Il pose souvent un problème de diagnostic positif clinique et histologique. La symptomatologie est généralement peu spécifique et la démarche étiologique repose sur la biopsie du cavum faite à l'examen endoscopique avec examen immuno-histochimique. Nous rapportons le cas d'un lymphome non hodgkinien avec atteinte du nasopharynx, l'analyse anatomopathologique est en faveur d'un lymphome malin non hodgkinien de phénotype B. Les aspects cliniques, radiologiques, histologiques et thérapeutiques sont décrits.

## Introduction

Les lymphomes représentent le troisième type de tumeur maligne au niveau de la tête et du cou (12%) après les carcinomes épidermoïdes (46%) et les carcinomes thyroïdiens (33%). L'incidence des lymphomes non hodgkinien est en augmentation depuis plusieurs décennies. Ils peuvent survenir à de multiples endroits de la tête et du cou, car la sphère ORL est riche en structures lymphoïdes [1]. Ils représentent un challenge diagnostic et thérapeutique [2]. Leur détection clinique intervient le plus souvent à un stade avancé en raison de la localisation anatomique profonde. Le diagnostic repose sur la biopsie avec étude immunohistochimique. Le traitement fait appel généralement à l'association chimio-radiothérapie. Nous présentons ici le cas d'une patiente atteinte d'un lymphome non hodgkinien de type B du cavum. Son pronostic rejoint celui des lymphomes d'autres localisations et demeure généralement bon.

## Patient et observation

**Figure 1:**
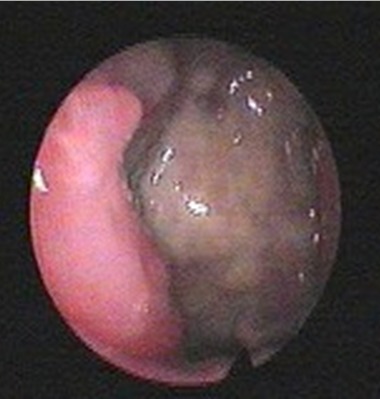
Image endoscopique de la tumeur du cavum

**Figure 2:**
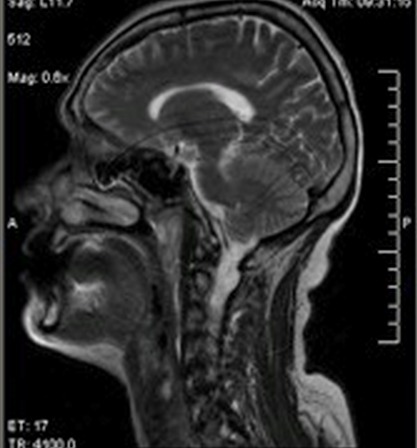
IRM du cavum: épaississement diffus de la paroi postérieure du rhinopharynx

**Figure 3:**
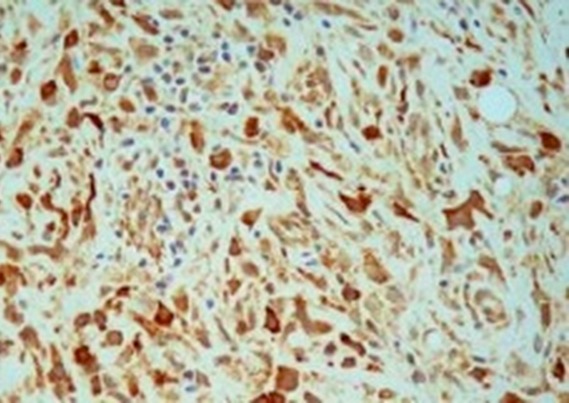
Histologie. Immunomarquage des antigènes CD 20

**Figure 4:**
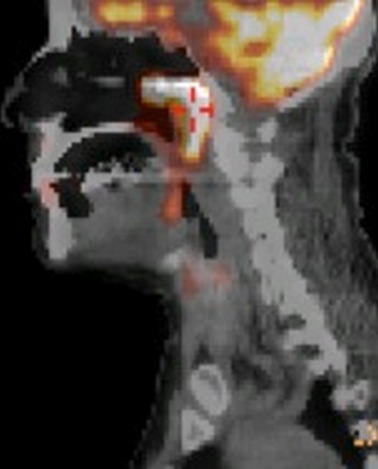
Image en TEP au 18F-Fluorodéoxyglucose: hyperfixations intenses au niveau du site tumoral rhinopharyngé (SUV max = 13,9) et d'une adénopathie du groupe II droit (SUV max = 6,9)

Mme A.K âgée de 57 ans, sans antécédents pathologiques notables, a présenté une obstruction nasale bilatérale prédominante à droite et évoluant depuis 12 mois avec voix nasonnée, associée à une odynophagie depuis 3 mois. L'examen clinique ORL, notamment la fibroscopie endonasale (Figure 1), a montré une tumeur violacée de la paroi rhinopharyngée postérieure para médiane droite qui s’étend depuis la partie inférieure de l'orifice tubaire droit jusqu’à l'oropharynx. Cette tumeur est inflammatoire et présente sur toute sa surface un dépôt pseudo-membraneux fibrinoïde. Des prélèvements locaux par écouvillons ont été effectués puis analysés en bactériologie. Tous les prélèvements étaient négatifs et l'IDR à la Tuberculine a été mesurée à 10mm. L'IRM cervico-faciale (Figure 2) a permis d'objectiver un épaississement diffus de la paroi postérieure du rhinopharynx, rehaussé par l'injection de Gadolinium et se prolongeant vers le bas par une formation fusiforme prévertébrale qui diminue le calibre des voies aéro-digestives supérieures, avec présence d'une adénopathie cervicale ovalaire du groupe II droit mesurant 17 mm. Le scanner thoracique n'a pas montré de foyer pulmonaire, et le bilan biologique était normal. Sous anesthésie générale, une endoscopie a été réalisée et plusieurs biopsies ont été effectuées au niveau de la formation tumorale rhinopharyngée. Ces biopsies, envoyées sans fixateur, ont fait l'objet d'une part d'une analyse histologique classique, et d'autre part d'une analyse après immuno-marquage. Cette analyse anatomo-pathologique a permit de mettre en évidence une muqueuse respiratoire très largement remaniée par un infiltrat polymorphe de cellules immunitaires dense et diffuse sans structure folliculaire avec des cellules de grande taille au contour nucléaire irrégulier; L'exploration immunohistochimique a trouvé que les fragments expriment la LCA, CD20 et CD79a et non EMA, CD3,CD15,CD30ni et CD45RO (Figure 3) posant le diagnostic d'un lymphome malin non hodgkinien à grande cellule B. La nature maligne de la lésion étant établie, une Tomographie par Emission de Positons au 18F-Fluorodéoxyglucose (TEP) a été demandée (Figure 4) afin de déterminer l'extension régionale et à distance du lymphome. Cet examen rapporte des hyperfixations intenses au niveau du site tumoral rhinopharyngé (SUV max = 13,9) mais aussi au niveau de l'adénopathie du groupe II droit (SUV max = 6,9), aucune autre lésion n'a été mise en évidence). La tumeur a ainsi été classée lymphome malin non hodgkinien à grandes cellules B stade I E (cavum) selon la classification d'Ann Arbor sans facteurs de pronostique défavorable [3, 4]. La patiente a pu bénéficier d'un traitement spécifique comportant une chimiothérapie avec 3 cures CHOP (Cyclophasphamide: 750 mg /m^2^, Adriamycine: 50 mg/m m^2^, Vincristine: 1,4 mg/m m^2^, Prédnisone) et irradiation de l'anneau de Waldeyer et des aires ganglionnaires cervicales de 45 Gy en étalement et fractionnement. La nasofibroscopie et le scanner du cavum ont montré la disparition de la lésion tumorale à la fin du traitement. Les contrôles périodiques endoscopique et radiologique après la fin du traitement ont montrés une rémission clinique et paraclinique. Le recul est de 8 ans.

## Discussion

Les lymphomes malins non hodgkinien LMNH sont les plus fréquentes des hémopathies malignes (15/100 000 habitants/an). Cette incidence a doublé en 20 ans. C'est la néoplasie qui a le plus augmenté en incidence après le mélanome. Sa localisation primitive au niveau du cavum est rare. Elle concerne moins de 10% des patients atteints de lymphome de la tête et du cou. Cette localisation particulière du lymphome se voit plus fréquemment en Extrême Orient et en Amérique du sud. La plupart des études publiées avaient tendance à l'inclure avec les autres lymphomes de la tête et du cou sans faire de différence; or le lymphome non hodgkinien du nasopharynx présente bien des particularités sur le plan épidémiologique, histologique et pronostique. Il peut se voir à tout âge mais plus souvent entre la cinquième et la sixième décennie et plus fréquemment chez le sexe masculin (sex-ratio de 3,6) [5] Les LMNH appartiennent aux lignées B ou T-natural killer (T/NK). Ils font partie des syndromes Lymphoprolifératifs matures. Le phénotype B prédomine pour les lymphomes non hodgkinien du cavum (60%) alors que le type T/NK prédomine au niveau de la cavité nasal. Le virus Epstein-Barr a été incriminé dans la genèse des lymphomes non hodgkiniens du nasopharynx mais uniquement de type T, car l'immunohistochimie ou la biologie moléculaire le révèle quasi constamment pour les lymphomes T mais non pour les B. L’étude immunohistochimique est indispensable afin de confirmer la nature lymphomateuse de la prolifération (expression de LCA) et de révéler le phénotype des cellules tumorales (expression de CD20 pour le phénotype B et de CD56 et CD45RO pour le phénotype T) [1, 3, 6]. Les signes cliniques d'appels du lymphome malin non hodgkinien sont non spécifiques identiques à ceux de toutes les tumeurs du cavum, rhinologiques d'une part avec obstruction nasale uni ou bilatérale, épistaxis, otologiques d'autre part en rapport avec un dysfonctionnement de la trompe d'Eustache, généralement il peut s'agir d'otite seromuqueuse [7]. Les adénopathies sont retrouvées habituellement dans la moitie des cas, elles sont fermes, mobiles et indolores. Les signes généraux sont retrouves dans 20% des cas et l'atteinte des paires crâniennes est extrêmement rare [7, 8]. L'examen endoscopique peut montrer une tumeur pâle ou violacée, molle, limitée au cavum ou étendue aux orifices tubaires ou aux choanes. Le plus souvent, le diagnostic se fait précocement (80%aux stades I et II) et le délai moyen est de 3 mois [3, 7]. Le bilan d'extension pour ces tumeurs comporte un bilan radiologique. La supériorité de l'IRM du cavum par rapport au scanner est établie notamment pour l'exploration des espaces profonds de la face. Cependant, la TDM garde sa place pour l’étude de la corticale osseuse de la base du crâne. L'exploration du cou jusqu'aux creux sus-claviculaires est recommandée a la recherche d'une extension. Le TEP-scanner est de plus en plus pratiqué pour la détection d'une extension régionale et à distance du lymphome, l'utilisation du TEP-scanner a modifié la prise en charge thérapeutique, soit en détectant des métastases à distance occultes (8%), soit en modifiant la classification ganglionnaire (25%), mais pas pour l'espace para- et rétropharyngé, la base du crâne et le sinus sphénoïdal où l'IRM garde sa supériorité. L'association IRM + TEP-scanner est souhaitable pour le bilan d'extension initial. Le TEP-scanner est, par ailleurs, d'utilisation croissante pour l’évaluation de la réponse au traitement. Sur le plan biologique, l'hémogramme et le dosage de Lacticodéshydrogénase (LDH), sont effectués de principe pour leur valeur pronostique [9].

Les LMNH présentent un certains nombres de facteurs pronostics (Tableau 1) qui permettent de prévoir le comportement clinique de la maladie et conduit à des indications thérapeutiques adaptées [4, 7]. Il n'existe pas de standard thérapeutique pour le lymphome non hodgkinien du cavum. Leur traitement rejoint celui des autres lymphomes non hodgkiniens [2]. Pour les lymphomes localisés sans facteurs de risque, le traitement associe une chimiothérapie (par trois cures de CHOP) à une radiothérapie avec obtention de bons résultats aussi bien sur le plan de la survie globale et du taux de rechute. Pour les lymphomes avec des facteurs de pronostic défavorable chez les sujets jeunes, les chimiothérapies les plus intensives sont indiquées que ce soit l'ACVBP (Doxorubicine, Cyclophosphamide, Vindésine, Bléomycine et Prednisone), le CHOEP (CHOP et Etoposide), le méga-CHOEP ou autre. Pour les patients plus âgés (61'70 ans), la chimiothérapie de type CHOP s'est imposée comme le traitement de choix du fait de son efficacité et surtout de sa toxicité acceptable. Pour les patients encore plus âgés (plus de 70 ans), l'utilisation d'une Anthracycline moins cardiotoxique, telle que la Farmorubicine (à la dose de 35 mg/m2 pour le mini-CEOP ou à la dose de 50 ou 70 mg/m2 pour le CEOP) s'est imposée dans les différents protocoles même si la toxicité est plus importante plus particulièrement chez les patients en mauvais état général. L'apport de l'intensification thérapeutique et du Rituximab pour les patients en situation de rechute chimiosensible est clairement démontré et représente une alternative pour ces malades [3, 7]. Quant à la radiothérapie, les volumes cibles comprennent l'anneau de Waldeyer et les aires ganglionnaires cervicales bilatérales à une dose de 45 à 55Gy en étalement et fractionnement classique. Actuellement, la radiothérapie devra être minimaliste (en termes de volume irradié et de dose) afin d’éviter les complications tardives L'utilisation de la radiothérapie conformationnelle, la radiothérapie avec modulation d'intensité et la radiothérapie guidée par l'image devrait minimiser la dose aux organes sains de voisinage [7, 10]. Les traitements sont réalisés dans un but curatif et il existe de nombreuses séquelles. Les toxicités tardives sont dominées par la xérostomie, le trismus, la fibrose cutanée, la toxicité auditive et à moindre degré un déficit neurocognitif. D'autres complications beaucoup plus rares peuvent être létales (rupture carotidienne notamment en situation de réirradiation) [10].

**Table 1:** Facteurs de mauvais pronostic d'un LMNH

Type	Mauvais pronostic
Age	> 60 ans
Stade Ann Arbor (AA)	III-IV
Etat général selon l’échelle de l'ECOG	>1
Taux de LDH (Lacticodéshydrogénase)	> normale
Nombre de sites extraganglionnaires	>1
Index de prolifération (Ki67)	> 20%
Lymphocytose	> 5 G/L
Réponse rapide au traitement	Mauvaise réponse après 2 cures

## Conclusion

Les lymphomes non hodgkinien du cavum représentent une entité rare. Si les présentations sont multiples, liées au stade, aux complications, aux localisations ou au type histologique, le diagnostic passera toujours par une biopsie permettant de typer le lymphome et de déterminer certains facteurs pronostiques liés à la cellule tumorale. Un bon résultat thérapeutique est tributaire d'un diagnostic précoce et d'une prise en charge multidisciplinaire. Les taux de survie globale sont de l'ordre de 40%, tous stades confondus.
